# Right ventricular function in transcatheter mitral and tricuspid valve edge-to-edge repair

**DOI:** 10.3389/fcvm.2022.993618

**Published:** 2022-10-12

**Authors:** Lukas Stolz, Philipp M. Doldi, Ludwig T. Weckbach, Thomas J. Stocker, Daniel Braun, Martin Orban, Mirjam G. Wild, Christian Hagl, Steffen Massberg, Michael Näbauer, Jörg Hausleiter, Mathias Orban

**Affiliations:** ^1^Medizinische Klinik und Poliklinik I, Klinikum der Universität München, Munich, Germany; ^2^German Center for Cardiovascular Research (DZHK), Partner Site Munich Heart Alliance, Munich, Germany; ^3^Herzchirurgische Klinik und Poliklinik, Klinikum der Universität München, Munich, Germany

**Keywords:** mitral regurgitation, tricuspid regurgitation, edge-to-edge repair, right ventricular function, MitraClip, PASCAL

## Abstract

Since transcatheter edge-to-edge repair (TEER) has become a valuable therapy in the treatment of both, mitral (MR) and tricuspid regurgitation (TR), the question of optimized patient selection has gained growing importance. After years of attributing rather little attention to the right ventricle (RV) and its function in the setting of valvular heart failure, this neglect has recently changed. The present review sought to summarize anatomy and function of the RV in a clinical context and aimed at presenting the current knowledge on how the RV influences outcomes after TEER for atrioventricular regurgitation. The anatomy of the RV is determined by its unique shape, which necessitates to use three-dimensional imaging methods for detailed and comprehensive characterization. Complex parameters such as RV to pulmonary artery coupling (RVPAc) have been developed to combine information of RV function and afterload which is primary determined by the pulmonary vasculature and LV filling pressure. Beyond that, TR, which is closely related to RV function also plays an important role in the setting of TEER. While mitral valve transcatheter edge-to-edge repair (M-TEER) leads to reduction of concomitant TR in some patients, the prognostic value of TR in the setting of M-TEER remains unclear. Overall, this review summarizes the current state of knowledge of the outstanding role of RV function and associated TR in the setting of TEER and outlines the unsolved questions associated with right-sided heart failure.

## Introduction

The clinical importance of transcatheter mitral and tricuspid valve repair has steadily increased within the past two decades. Despite a growing number of transcatheter techniques, edge-to-edge valve repair (TEER) remains the most commonly used clinical procedure until today and has been proven to improve prognosis in case of mitral regurgitation (MR) (1, 2). The health and socio-economic importance of these procedures is immense, since both tricuspid (TR) and MR have an increasing prevalence, especially in elderly patients, and are associated with high rates of heart failure hospitalizations (HHF), morbidity, and mortality (3–5).

While the beneficial effect of mitral valve transcatheter edge-to-edge repair (M-TEER) has been proven in randomized controlled trials (1, 6), studies comparing tricuspid TEER (T-TEER) to diuretic treatment alone are still ongoing. So far, observational studies reported high rates of procedural TR reduction and suggest low mortality rates in propensity-matched analysis, especially compared to conventional surgical treatment (7–9).

Historically, both the right ventricle (RV) and the tricuspid valve, often referred to as the “forgotten valve” have received less scientific attention than the left ventricle (LV). This could be partly explained by high procedural/perioperative and short-term mortality rates for right heart surgical procedures in this high-risk population. Another reason was the lack of comprehensive three-dimensional imaging models that could reflect the complex and irregular anatomy of the RV (10). As this has changed in parallel with the advance of transcatheter repair techniques, the geometry and function of the right ventricle (RV) have increasingly become the focus of research in the field of TEER. The right ventricle plays an important role in the complex interplay between the left ventricle (LV), pulmonary and systemic circulation. Understanding function and geometry of the RV with echocardiographic methods remains challenging and is subject to constant technical and methodological progress.

The aim of this review was to provide a comprehensive overview about (1) RV anatomy, (2) RV function, and (3) the role of RV geometry and function in the context of mitral and tricuspid TEER.

## Anatomy of the right ventricle and tricuspid valve

**FIGURE 1:**
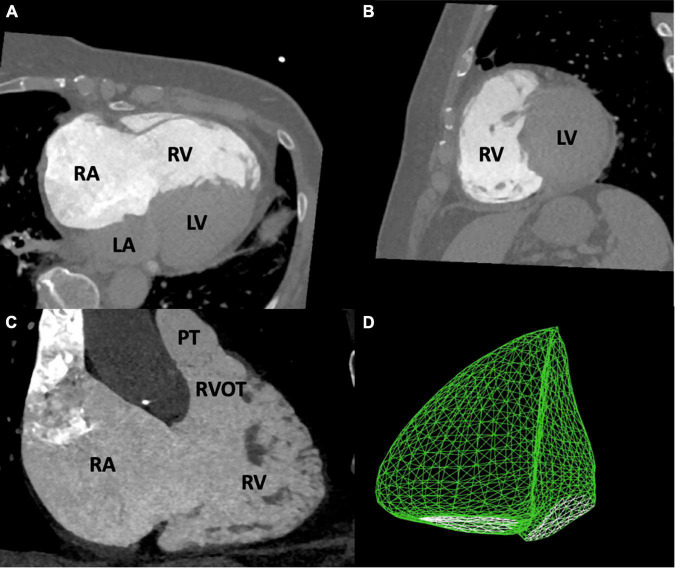
Anatomy of the right ventricle. This figure shows images of the right heart derived from multidetector computed tomography **(A–C)** and three-dimensional echocardiography **(D)**. The RV is crescent shaped in an axial **(A,C)** and triangular in a lateral view **(B)**. LA, left atrium; LV, left ventricle; PT, pulmonary trunk; RA, right atrium; RV, right ventricle; RVOT, right ventricular outflow tract.

The RV is the most anteriorly located chamber of the human heart (11). Its shape is difficult to approach geometrically but is commonly referred to as being crescent shaped in an axial and triangular in a lateral view (Figure 1) (11, 12). Usually, the right-convex interventricular septum is assigned to the left ventricle. While being 10% larger than the LV on average in volume, the RV muscular mass is significantly lower due to low pressure conditions and a relatively thin free wall (12–14). Anatomically it can be divided into three distinguishable regions, the inlet, apex, and outlet. The RV inlet includes the tricuspid valve (TV), the chordae tendineae and extends to a more variable number of papillary muscles compared to the LV (11, 12). The apex consists of much more trabecularized myocardium compared to the LV. The outlet (also described as infundibulum or conus) forms the complete muscularly shaped outflow tract of the RV (RVOT) (11, 12). Three prominent muscle bands can be delineated within the RV. Together, the parietal band and the infundibular septum are described as the crista supraventricularis and separate RVOT and TV (11–13, 15). The septomarginal band is described as Y-shaped which is connected to the medial papillary muscle with one arm and to the subpulmonary infundibulum with the other arm (15). The trunk of the Y continues into the moderator band which contains a prominent fascicle of the right bundle of the conduction system (12, 15).

The TV separates the right atrium (RA) from the RV and consists of leaflets (endocardial duplications), the annulus, papillary muscles, and chordae tendineae (16, 17). Contrary to what the name suggests, the TV does not always consist of three leaflets but can be subject to considerable anatomical variation (18). The TV is located further apically than the MV and does not have any fibrous connection to the pulmonary valve (PV) (19). The septal leaflet is characteristically connected to the interventricular septum by several direct small chordae tendineae (15). The septal and anterior leaflets are usually supported by a small medial (septal) papillary muscle. The anterior and posterior leaflets additionally are attached to a comparably large anterior papillary muscle originating from the moderator band (15). The posterior leaflet receives support from a variety of papillary muscles arising from the diaphragmatic RV wall which are sometimes summarized as the posterior papillary muscle (15, 16).

## Function and dynamics of the right ventricle

To describe RV function and its contraction patterns, it is crucial to understand the RVs myoarchitecture. The muscular wall of the RV is arranged in two layers. The outer (superficial) layer of cardiomyocytes is arranged circumferentially to the TV annulus. Toward the RV apex, the fibers turn slightly oblique and continue into the superficial layer of the LV myocardium (11). The deep layer of myocytes is longitudinally aligned (12). Contraction of the RV usually begins at the inlet and apex, followed by the outlet approximately 25–50 ms later (11, 12). Some authors even describe the RV’s contraction pattern as “peristalsis-like” (20). Compared to the LV, the RV has significantly fewer oblique fibers, allowing longitudinal contraction to account for a large proportion of ventricular function (12). While circular fibers lead to an inward movement of the RV, the connection of RV and LV myofibers and the common interventricular septum lead to a significant proportion of RV function being attributable to LV contraction (12). Blood flow through the RV is believed to be relatively well streamlined within the inflow and curved apex until it becomes helical when entering the pulmonary circulation through the outflow and PV (12, 21, 22).

The overall systolic function of the RV is a complex interplay of preload (systemic venous return), contractility, and afterload (pulmonary pressure). Due to the large surface compared to the RV volume and a relatively thin wall, according to Laplace’s law, the RV can adopt to a broad spectrum of preload alterations but struggles with rapid changes in afterload (23). Rapid changes in pre- or afterload lead to dilation of the RV which normalizes once contractility has adequately been increased (12, 23).

The TV annulus shows a unique saddle-shaped anatomic configuration with the most atrial located point in the antero-posterior direction and the most ventricular located point in the medio-lateral direction (24). It is a highly dynamic structure within the cardiac cycle and depending on loading conditions (19). Of note, the tricuspid annulus is apically displaced compared to the MV annulus. In patients with functional TR, the TV annulus dilates in an lateral and posterior direction toward the RV free wall which leads to flattening and rounding of the annular geometry (24).

## Right ventricular/tricuspid valve geometry and function in the context of transcatheter edge-to-edge repair

### Routine echocardiographic parameters of right ventricular function

Since echocardiographic assessment of right ventricular function is challenging, routine RV function parameters [tricuspid annular plane systolic excursion (TASPE), RV fractional area change (RVFAC)] are subject to a variety of limitations. TAPSE is usually measured using the M-mode in an apical four chamber view and represents the longitudinal shortening of the tricuspid annulus and hence RV in one plane. Even though longitudinal contraction significantly contributes to the overall RV function, it does not consider shortening in the other two dimensions (25). Further, measurement of TAPSE is dependent of proper M-mode alignment which can be challenging in case of small echo windows (26). In contrast, RVFAC also respects the above-mentioned radial contraction component of the RV as it is derived from RV end-systolic and end-diastolic areas. Measuring RVFAC highly depends on the image plane acquired and shows comparably low interobserver agreement (26). In order to overcome the above-mentioned limitations, novel parameters and three-dimensional echocardiography were introduced.

### Right ventricular to pulmonary artery coupling

**FIGURE 2:**
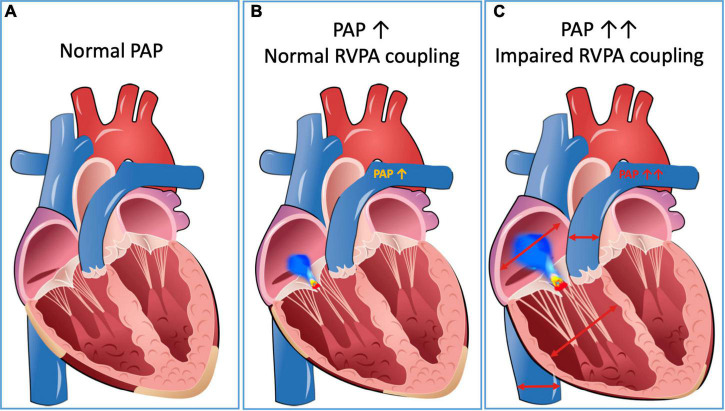
RV-PA interdependency. Panel **(A)** represents a compensated situation with normal PAP and RV function. In panel **(B)**, RVPAc is intact, and the RV can compensate increasing afterload by mild dilation. In panel **(C)**, RVPA uncoupling occurs, which is accompanied by significant TR and dilation of the IVC. IVC, inferior vena cava; PAP, pulmonary artery pressure; RV, right ventricle; RVPA, right ventricular to pulmonary artery; TR, tricuspid regurgitation.

Recently, a parameter called RV to pulmonary artery coupling (RVPAc) was introduced to quantify the close interdependency of the RV and its afterload (27, 28). Under physiological conditions, RVPAc is intact, and the function of the RV can adapt to the changes in pulmonary pressure conditions. In addition to the Frank Starling mechanism, neurovegetative and humoral mechanisms also contribute to this (29). In the case of RVPA uncoupling the afterload exceeds a certain threshold, and the RV cannot adequately increase its contractility (30). As a result, pathological dilatation of the RV occurs, often accompanied by the development of TR, as well as reduced RV function resulting in systemic congestion and secondary organ dysfunction (31, 32) (Figure 2).

Echocardiographically, RVPAc can be quantified as a ratio of, in principle, any RV functional parameter (e.g., TASPE, RV fractional area change RVFAC, RV longitudinal strain RVLS) and pulmonary artery pressure (PAP). In 2021, a large European multicentric registry of patients who underwent M-TEER for severe SMR found RVPA uncoupling as defined by a TAPSE/sPAP ratio <0.274 mm/mmHg to be associated with significantly impaired 2-year survival rates (33). A subanalysis of the COAPT (Cardiovascular Outcomes Assessment of the MitraClip Percutaneous Therapy for Heart Failure Patients with Functional Mitral Regurgitation) study defined RVPA uncoupling as RVLS of the free RV wall/sPAP ratio. A RVPA value of <0.5%/mmHg was associated with significantly higher rates of mortality or heart failure hospitalizations in both the treatment (M-TEER + GDMT) and control (GDMT only) group (30). Of note, M-TEER improved outcome of SMR patients independent of RVPA uncoupling (30). In the meantime, the prognostic value of RVPAc has also been shown in patients with primary mitral regurgitation (PMR) (34).

In patients with severe MR, RVPA uncoupling, and thus biventricular failure is most likely the consequence of long standing regurgitant blood flow across the MV in systole and development of secondary pulmonary hypertension. In patients undergoing T-TEER for severe TR, RVPA uncoupling may occur if the etiology of TR is secondary to any kind of left sided disease.

Recently, a large observational study identified RVPA uncoupling defined as TAPSE/sPAP ratio <0.406 mm/mmHg as independent predictor for 1-year all-cause mortality after T-TEER for severe TR (35). sPAP is usually approximated by using transtricuspid pressure gradients and width of the inferior vena cava. Even though the calculated cut-off in the studied T-TEER population had predictive value, the absolute values should be interpreted with caution. Especially in severe, massive or torrential TR with large coaptation gaps and high EROA, rapid systolic pressure equalization between RA and RV occurs leading to false low transtricuspid pressure gradients and underestimation of sPAP (36). Future studies are needed to evaluate whether calculating RVPA coupling should rather be performed using invasive PAP values in the setting of TR and T-TEER.

### Right ventricular contraction patterns

Right ventricular contraction is a complex process in both spatial and temporal dimensions. Using up-to-date imaging protocols in echocardiography and cardiac magnetic resonance (CMR) have resolved the technical challenges in visualizing and adequately measuring RV function. These methods have provided important insights into the role of the RV for atrioventricular regurgitation.

A recent study focused on the prognostic impact of RV contraction patterns in the setting of T-TEER for severe TR using CMR imaging (37). The authors distinguished three different contraction patterns. Pattern I: Preserved longitudinal (TAPSE ≥ 17 mm) and preserved global RV function (RVEF > 45%). Pattern II: Impaired longitudinal (TAPSE < 17 mm) and preserved global RV function (RVEV > 45%). Pattern III. Impaired longitudinal (TAPSE < 17 mm) and global RV function (RVEF ≤ 45%). Patients who underwent T-TEER and presented with RV contraction pattern III had a significantly higher risk of death or HHF. The authors conclude that TAPSE alone is not sufficient to characterize RV function in T-TEER patients, because especially in the presence of pressure overload circumferential RV function increases by hypertrophy of the outer myocardial layer to compensate the functional decline in a longitudinal direction (37). Especially in combination with additional volume overload, the RV dilates and finally loses its ability to compensate RV function which may lead to right heart decompensation and impaired survival (37). Of note, RVEF < 45% itself was a strong and independent predictor for the combined endpoint.

### The value of three-dimensional echocardiography in transcatheter edge-to-edge repair

Within the past few years, three-dimensional echocardiography (3DE) has emerged as state-of-the-art imaging technique, as it is better suited to the geometric and functional complexity of the RV than biplane methods (Figure 1D) (38). A recent study evaluated the prognostic value of 3DE-derived RV function in patients undergoing T-TEER (39). In agreement with the cut-off established in CMR studies on RV function (RVEF < 45%) (37), the authors were able to identify an RVEF value <44.6% as a negative prognostic predictor for postinterventional survival in T-TEER patients (39). Additionally, comparative studies have confirmed this agreement of RVEF derived from CMR and 3DE measurements (40, 41).

As recent data show, T-TEER treatment not only leads to a significant reduction in TR, but was also associated with RV reverse remodeling (RVRR) (42). RV dimensions as well as tricuspid annular diameter (TAD) significantly decreased within the first 6 month after treatment. Of note, TAPSE remained unchanged while RVEF declined significantly. The authors interpreted this phenomenon as “ejection fraction normalization” due to the reduction of the regurgitant blood flow across the tricuspid valve and subsequent increase in effective forward RV stroke volume (42). Beyond that, RVPAc improved significantly after T-TEER (42). RVRR was even associated with better prognosis after T-TEER. Now that M-TEER treatment is also known to reduce concomitant TR, further studies are needed to investigate a possible RVRR in this patient population.

## Anatomic variability of the tricuspid valve in the context of transcatheter edge-to-edge repair

**FIGURE 3:**
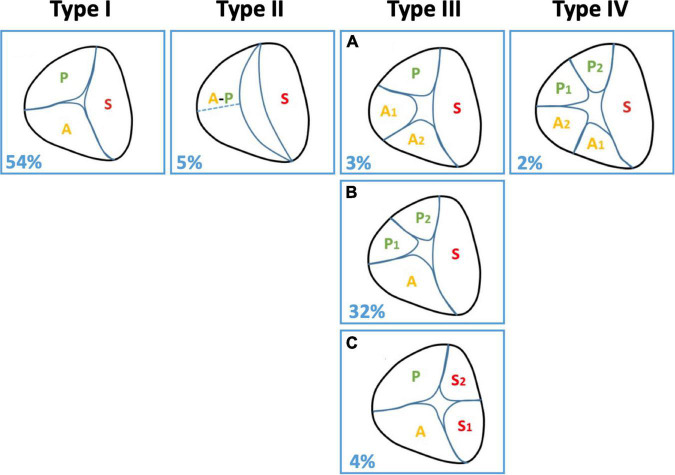
TV anatomic patterns. This figure illustrates the different types of TV anatomy. A, anterior; P, posterior; S, septal; TV, tricuspid valve.

A crucial point in treatment planning and device selection in high-grade TR is the anatomy of the TV, which is often challenging due to the high variability. In 2021 Hahn et al. presented a systematic classification of different TV morphologies (18). In this context, they proposed to distinguish between six different anatomical configurations (Type I, II, IIIA-C, IV) (Figure 3) (18). While Type I showed a “normal” leaflet configuration with one anterior, one septal and one posterior leaflet, in Type II fusion of the anterior and posterior leaflet led to a “two-leaflet configuration” of the TV. In Type III, one leaflet was subdivided and leads to a “four-leaflet-configuration” (IIIA: Anterior leaflet divided; IIIB: Posterior leaflet divided; IIIC: Septal leaflet divided). Finally Type IV TV was defined as having five leaflets (18). In descending order, the different anatomic subtypes were observed with varying frequency (Type I: 54%, Type IIIB: 32%, Type II: 5%, Type IIIC: 4%, Type IIIA: 3%, Type IV: 2%) (18).

In an additional study, the authors investigated the influence of this classification on outcome after T-TEER (43). They observed no significant outcome differences regarding the number of implanted TEER devices, TR reduction, NYHA functional class, HHF and 1-year all-cause mortality (43). In contrast, Sugiura et al. investigated the impact of a three vs. four leaflet anatomy of the TV on residual TR after T-TEER (44). The latter was observed in about 30% of patients and showed association with increased rates of postprocedural residual TR ≥ 3+ (44). Recently, a retrospective study outlined the prognostic importance of the “leaflet-to-annulus-index” (LAI) on procedural TR reduction after T-TEER (45). The LAI is calculated as anterior leaflet length plus septal leaflet length divided by the septolateral tricuspid annulus diameter and was a significant and independent predictor as postprocedural TR ≥ 3+ (45). Even though the overall rates of TR reduction after T-TEER are excellent, TV anatomy might impact procedural outcomes.

## The role of tricuspid regurgitation in mitral valve transcatheter edge-to-edge repair

Development of pulmonary hypertension and right ventricular dysfunction (RVD) in patients undergoing M-TEER might be associated with concomitant TR. Recent studies focused on the change in TR severity after M-TEER by hemodynamic pulmonary circulatory relief after MR reduction. Successful M-TEER was associated with modest reduction in TR severity as early as 1 month after procedure (46, 47). Nevertheless, in a significant proportion of patients, TR remains stable or even worsens. Several retrospective observational studies sought to identify predictors for worsening TR after M-TEER. Identified predictors were atrial fibrillation, the degree of residual postprocedural MR, TAD and less procedural sPAP reduction (47, 48). Consequently, effective reduction of MR, as well as preserved RV dimensions seem to be important prerequisites for reduction of concomitant TR. Although studies have shown promising success rates of simultaneous mitral and tricuspid transcatheter repair (M/T-TEER) (9), the question of optimal patient selection for M/T-TEER and its added benefit remains open.

Furthermore, it is controversial whether moderate or advanced TR has independent prognostic importance in patients undergoing M-TEER for severe MR. A subanalysis of the COAPT trial sought to assess on outcomes in patients with HF and severe secondary MR in both the treatment and control-group. Of note per protocol, patients with TR requiring surgery or transcatheter treatment were excluded. Overall, 15.4% presented with moderate and, 0.8% with moderate-to-severe and 0.2% with severe TR. Interestingly, TR ≥ 2+ was a significant independent predictor for the combined endpoint (HHF/2-year mortality) in the GDMT, but not in the M-TEER group (49). Of note, due to the fact that patients with more severe concomitant TR presented with smaller LV dimensions while having comparable LV function and RV dimensions, the authors hypothesize that these individuals represent a certain MR phenotype with combined pre- and postcapillary pulmonary hypertension (49). In contrast to the controlled conditions of the COAPT study, under “real-world” conditions also patients with severe TR are treated. To what extent the results are applicable to such a patient population is unclear at the moment. Real-world observational studies are inconclusive about the prognostic impact of high-grade TR after M-TEER (50–52).

## Summary, gap of evidence and conclusion

The “renaissance” of the RV and TV in cardiovascular medicine and research has clearly a significant impact on the dynamic field of transcatheter edge-to-edge repair. For both mitral and tricuspid valve repair, the RV is gaining importance in clinical decision making due to its eminent pathophysiologic and therefore prognostic role.

The interplay of RV, MV, and TV and their respective extravalvular structures needs a detailed preprocedural multimodal imaging evaluation for treatment planning. This is of utmost importance for the TV, as anatomic and functional variability is high (18). Valve imaging is still a relatively novel field with recent advances in 3D assessment. These have improved our understanding of their valve anatomy and have contributed to the ongoing success of TEER. While CMR is currently the “state-of-the-art” imaging modality for MR and RV assessment, its wide-spread availability is limited. For TR, the best imaging modality has yet to be defined. Therefore, 3D echocardiography of the RV is rapidly evolving and getting closer to the gold standard in terms of prognostic information and reliability, as it is the primary imaging modality in most transcatheter-treated patients.

Optimizing patient selection for T-TEER or combined M/T-TEER is an important question to be resolved in the future. Although some observational studies have addressed this issue, randomized data are urgently needed. In fact, randomized trials of T-TEER are underway, and will confirm or refuse the prognostic impact of e.g., proposed cut-off values of RV dysfunction (37, 39). But even in the case of valid prognostic value, the decision to refuse a T-TEER treatment in case of severe RV dysfunction is not based on one parameter but on the combined knowledge expressed by the heart team.

For combined M/T-TEER, it is currently unknown whether an approach similar to surgery has added benefit over isolated TEER or a staged procedure. In the surgical field, current guidelines recommend concomitant tricuspid surgery for severe TR when primary left-sided valve surgery is indicated. In case of mild or moderate TR, certain anatomic parameters have to be taken into account for this concomitant surgical approach. For the transcatheter approach, there is no such recommendation, as concise data is missing. Potentially, RV and LV function could give us the answer which patients needs concomitant, staged, or isolated treatment with a meaningful clinical benefit in each of the latter cases.

Even though numerous retrospective studies were performed to shed light into the world of T-TEER, until today, there have been no randomized-controlled studies which have shown the benefit of T-TEER and optimal medical therapy (OMT) vs. OMT alone. Currently, four large, randomized trials are ongoing in order to close this gap in evidence (TRILUMINATE, CLASP II TR; TRI-FR; TRIC-I-HF). Beyond that, it remains unclear which patients with concomitant mitral and tricuspid regurgitation need to be treated simultaneously and in which patients’ treatment of MR alone might be sufficient.

In conclusion, we believe that RV function is of key prognostic importance in patients undergoing TEER and its evaluation needs to be performed using state of the art 3DE technologies in order to comprehend the RVs anatomical and functional complexity. We believe that the journey toward a comprehensive understanding of RV function and hemodynamics has only begun but can further improve the quality of TEER treatment through optimized patient selection.

## Author contributions

LS and MatO wrote the preliminary version after conceptual development. CH, DB, JH, LW, MarO, MN, MW, PD, SM, and TS edited the text and figures according to their expertise. All authors contributed to the article and approved the submitted version.
